# Description of the targeted water supply and hygiene response strategy implemented during the cholera outbreak of 2017–2018 in Kinshasa, DRC

**DOI:** 10.1186/s12879-020-4916-0

**Published:** 2020-03-18

**Authors:** Didier Bompangue, Sandra Moore, Nadège Taty, Benido Impouma, Bertrand Sudre, Richard Manda, Thierno Balde, Franck Mboussou, Thierry Vandevelde

**Affiliations:** 1 Ministry of Health, Kinshasa, Democratic Republic of the Congo; 2 Faculty of Medicine, University of Kinshasa, Kinshasa, Democratic Republic of the Congo; 3 Laboratory Chrono-Environnement, UMR 6249, University of Bourgogne Franche-Comté, Bourgogne Franche-Comté, France; 4 Veolia Foundation, Aubervilliers, France; 5 World Health Organization, African Regional Office, Brazzaville, Republic of, Congo

**Keywords:** Cholera, Vibrio cholerae, Kinshasa, Democratic Republic of the Congo, Water supply, Water treatment, Hygiene promotion, Outbreak response, Case cluster-targeted interventions

## Abstract

**Background:**

Rapid control of cholera outbreaks is a significant challenge in overpopulated urban areas. During late-2017, Kinshasa, the capital of the Democratic Republic of the Congo, experienced a cholera outbreak that showed potential to spread throughout the city. A novel targeted water and hygiene response strategy was implemented to quickly stem the outbreak.

**Methods:**

We describe the first implementation of the cluster grid response strategy carried out in the community during the cholera outbreak in Kinshasa, in which response activities targeted cholera case clusters using a grid approach. Interventions focused on emergency water supply, household water treatment and safe storage, home disinfection and hygiene promotion. We also performed a preliminary community trial study to assess the temporal pattern of the outbreak before and after response interventions were implemented. Cholera surveillance databases from the Ministry of Health were analyzed to assess the spatiotemporal dynamics of the outbreak using epidemic curves and maps.

**Results:**

From January 2017 to November 2018, a total of 1712 suspected cholera cases were reported in Kinshasa. During this period, the most affected health zones included Binza Météo, Limeté, Kokolo, Kintambo and Kingabwa. Following implementation of the response strategy, the weekly cholera case numbers in Binza Météo, Kintambo and Limeté decreased by an average of 57% after 2 weeks and 86% after 4 weeks. The total weekly case numbers throughout Kinshasa Province dropped by 71% 4 weeks after the peak of the outbreak.

**Conclusion:**

During the 2017–2018 period, Kinshasa experienced a sharp increase in cholera case numbers. To contain the outbreak, water supply and hygiene response interventions targeted case households, nearby neighbors and public areas in case clusters using a grid approach. Following implementation of the response, the outbreak in Kinshasa was quickly brought under control. A similar approach may be adapted to quickly interrupt cholera transmission in other urban settings.

## Background

Cholera is an acute diarrheal disease caused by ingesting water or food contaminated with toxigenic forms of *Vibrio cholerae* [1]. Once an individual contracts cholera, subsequent disease transmission is associated with limited access to clean drinking water and poor sanitation [1, 2]. The disease continues to represent a global public health concern, especially in Sub-Saharan Africa [3].

To stem this public health threat, the Global Task Force for Cholera Control (GTFCC) has endorsed a call to action in 2017 with the plan “Ending Cholera – A Global Roadmap to 2030” [4]. The Ending Cholera Roadmap aims to put an end to cholera epidemics in up to 20 countries and reduce cholera-related deaths by 90% by the year 2030 [4]. The multisectoral approach involves early detection and quick response to contain outbreaks. However, rapid control of cholera outbreaks can be a major challenge in urban settings, where cholera case numbers can quickly increase [2, 5]. Once cholera outbreaks expand in urban areas, cholera has often eventually spread to linked regions within the country or across international borders when infected individuals travel [6–8]. As a result, effective strategies to control cholera outbreaks in urban settings may play a major role in the control and prevention of the disease on a local, national and regional level.

Over the past two decades, the Democratic Republic of the Congo (DRC) has borne a significant proportion of the global cholera burden. Between 2010 and 2017, the DRC reported approximately 220,000 suspected cholera cases, accounting for 25% of all cholera cases notified in Africa [3]. Cholera is considered endemic in the African Great Lakes region of eastern DRC, where cholera cases have been consistently reported for over 25 years [9, 10]. By contrast, cholera outbreaks have only intermittently occurred in the western DRC provinces, including the capital Kinshasa [10–12]. Although Kinshasa has only been sporadically affected by the disease, the city remains vulnerable to outbreaks. In 2011, an epidemic spread outside of the cholera-endemic zone in eastern DRC and along the Congo River towards Kinshasa Province [12, 13]. The cholera outbreak quickly spread throughout Kinshasa [9] and continued for 116 weeks, resulting in 2144 cases (case fatality rate (CFR) 2.3%) [12].

In recent years, cholera case numbers have increased in the DRC [3, 10]. The epidemic of 2017 represented the largest cholera epidemic to affect the country since 1994, with approximately 56,000 cholera cases reported nationwide [3]. During this time, cholera outbreaks again spread outside of the cholera-endemic zone in eastern DRC and into western provinces, including Kinshasa. Between April 2016 and March 2018, Kinshasa experienced three cholera outbreaks of increasing intensity, with weekly case numbers peaking in December of 2017.

In this study, we describe the targeted water and hygiene response strategy designed to stop cholera transmission in Kinshasa during the outbreak in late 2017. We also performed a preliminary community trial study to assess the temporal pattern of the outbreak, in terms of number of cases per health zone and Kinshasa Province overall, before and after response interventions were implemented.

## Methods

### Study design and site

In this report, we describe the first implementation of the cluster grid response strategy carried out in the community during the cholera outbreak in Kinshasa, in which water and hygiene interventions were carried out in cholera case clusters. We also performed a preliminary community trial study to assess the temporal pattern of the outbreak in Kinshasa, in terms of number of cases per health zone and Kinshasa Province overall, before and after response interventions were implemented. Cholera surveillance databases from the Ministry of Health were analyzed to assess the spatiotemporal dynamics of the outbreak using epidemic curves and maps.

**Fig. 1:**
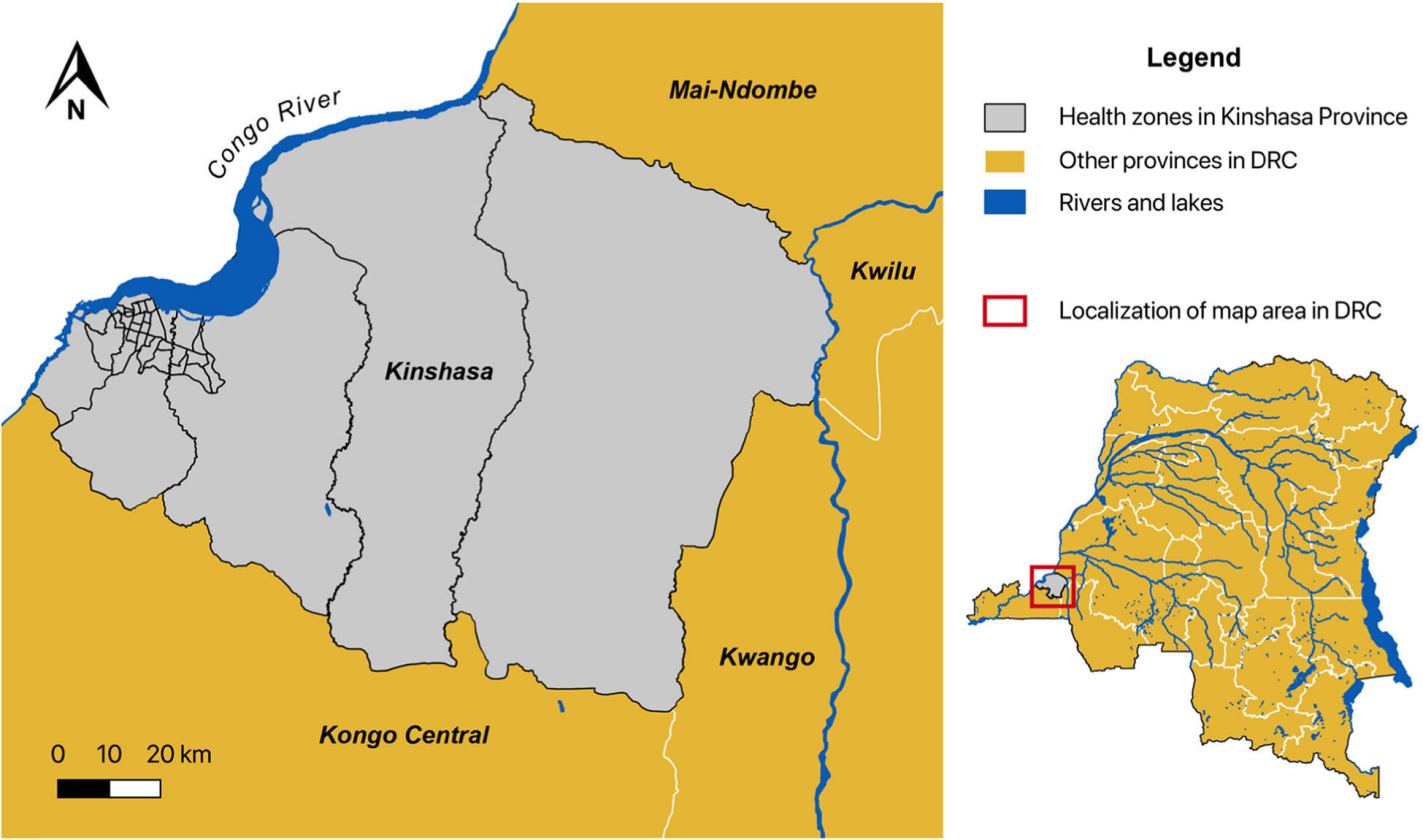
Map of study area: Kinshasa Province. DRC, Democratic Republic of the Congo

Kinshasa Province is one of 26 provinces in the DRC and is coterminous with the national capital. The city-province is divided into 35 administrative health zones. Kinshasa is located in the far west of the country on the banks of the Congo River (Fig. 1). The province covers approximately 9965 km^2^, with an estimated population of nearly 12 million. Infrastructure measures in the city have not kept pace with urbanization and the increasing population [14]. As a result, neighborhoods have been established in flood-prone areas where water drainage is a challenge, thus increasing the risk and severity of flooding, especially during heavy rains in November and April [15].

### Surveillance data sources

The National Integrated Disease Surveillance and Response System was established in 2000 by the DRC Ministry of Health in conjunction with the World Health Organization (WHO). The Integrated Disease Surveillance and Response System targets thirteen infectious diseases with epidemic potential (including cholera) for passive surveillance [16]. In each cholera treatment center (CTC), suspected cases and deaths due to moderate or severe cholera infection are documented via line list [17], which includes the patient’s address, age, sex, date of admission, date of onset, travel history during 14 days prior to symptom onset, and observation of any other individuals with diarrhea living in the patient’s home. Trained Ministry of Health officials aggregate and anonymize these data at the health zone level and report the data to the Ministry of Health in Kinshasa on a weekly basis.

### Cholera case definition

According to WHO policy, a suspected case of cholera is defined as “any person 2 years of age or older in whom acute watery diarrhea with or without vomiting develops” during a cholera outbreak [18]. The age limit is increased to 5 years and older during inter-epidemic periods to reduce the number of false positives. At the beginning of an outbreak, five to ten stool samples from each health zone are laboratory-confirmed through identification of *Vibrio cholerae* in culture. Subsequent cases of acute watery diarrhea in the same geographic region are presumed to be cholera.

### Management and analysis of epidemiological data

Secondary data was extracted from surveillance databases organized by staff of the National Program for Cholera Elimination and Diarrheal Disease Control (Programme National d’Elimination du Choléra et de lutte contre les Maladies Diarrhéiques [PNECHOL-MD]). The database was verified for consistency and analyzed to determine weekly case numbers per health zone using Microsoft Excel. Epidemic curves per health zone were generated to assess the temporal evolution of the outbreak in Kinshasa as well as outbreaks in each affected health zone in Kinshasa Province, covering the period week 1 of 2017 to week 45 of 2018 (the epidemic curve shows cases starting from week 15 of 2017 because few cases were reported earlier in the year). Total weekly case numbers per health zone and in Kinshasa Province were also calculated to assess the temporal pattern of the outbreak before and after response interventions were implemented. The total suspected cholera case numbers reported in each health zone from November 1st 2017 to March 31st 2018 were used to represent the geographic distribution of cholera cases during the main outbreak period shown in Fig. 4.

### Cartography

The maps of Kinshasa and the DRC were generated using QGIS V3.4.3 Madeira with shapefiles provided by the DRC Ministry of Health (DRC health zones, DRC provinces, rivers and lakes). Additionally, shapefiles of Republic of the Congo administrative boundaries and transportation network features (rail and road) were retrieved from DIVA-GIS (http://www.diva-gis.org/gdata). The GPS coordinates of the CTCs were provided by the Kinshasa Ministry of Health.

### Precipitation data

Precipitation levels were derived from the Climate Hazards Group InfraRed Precipitation with Station (CHIRPS) dataset (product: Daily UCSB CHIRPS v2p0 daily-improved global 0p25). The CHIRPS precipitation data is a 30-year quasi-global rainfall dataset supported by the University of California at Santa Barbara (CA, USA). Daily values were extracted and aggregated by health zone (R environment for statistical computing and graphics). Spatial aggregation from gridded data at the province level for Kinshasa was carried out using R. Daily precipitation levels (mm) were then aggregated by week using Microsoft Excel.

### Field visits

Field visits were conducted by joint investigation teams composed of representatives of the PNECHOL-MD, Provincial Health Directorates and community agents in each affected health zone. Investigation teams met with local surveillance departments and health facilities. Information was collected concerning potential sources of infection, risk factors and possible links between cases [17]. In each case cluster, the investigation teams also evaluated local demographic data, WASH (water, sanitation and hygiene) indicators and other factors that may play a role in cholera dynamics [19]:

- Local demographic data (number of people per household, occupation and workplace of adults in the household).
- Factors contributing to amplification or persistence of an outbreak: high population density, potentially contaminated drinking water sources, poor water quality, poor sanitation (open defecation, broken sewer pipes, etc.), poor food hygiene, crowded or high-risk gathering places (markets, transportation hubs, schools, waterbodies used for bathing, etc.), and flooding.

### Description of the targeted response using the cluster grid strategy

The main objective of the cluster grid response strategy was to quickly target case clusters (including affected households and at-risk populations in the community), in which interventions focused on emergency water supply, household water treatment and safe storage, home disinfection and hygiene promotion [19–23].

To inform and guide the targeted response, the epidemiological data was analyzed to first identify the most affected health zones (reporting more than 10% of the total suspected cholera cases during the previous three-week period). Each new affected health zone that experienced a laboratory-confirmed outbreak and displayed risk factors that may trigger an increase in cases according to field investigations was also included in the response. As a result, five health zones (Binza Météo, Limeté, Kintambo, Kingabwa and Bumbu) were selected for targeted water supply and hygiene interventions. Over the course of the outbreak, daily case admission trends, including origin of patients, were monitored to assess the epidemiological evolution of the outbreak in real time and adjust response activities accordingly.

To target case clusters within each of the five health zones, the line lists of suspect cases were consulted in the CTCs to obtain the address of patients admitted over the previous 14-day period (CTCs were established in Limeté (Pakadjuma) and Binza Météo (Camp Luka)).

**Fig. 2:**
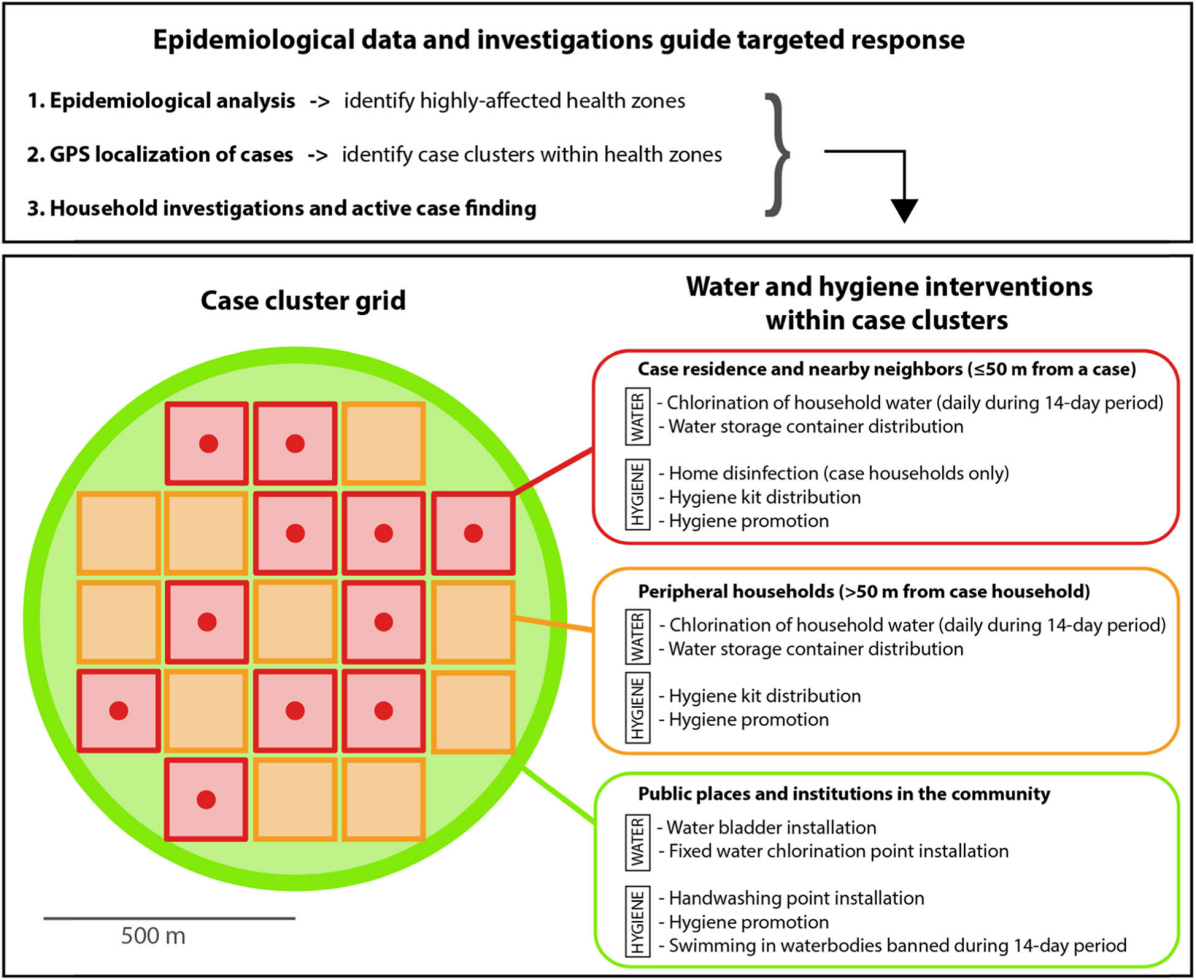
Schematic diagram of the cluster grid response strategy. The case cluster is shown in green, case residences are represented by red dots, nearby neighbors (≤50 m from a case household) are represented in red squares and peripheral neighbors (> 50 m from a case household, within the case cluster) are represented in orange squares

A response team then visited each patient residence to obtain GPS coordinates of the case households. The location of the most recent cases (< 14 days) were mapped, and a circle (500-m radius) was delineated around each cluster, which was then subdivided into a grid. Each grid unit represented an average of 20–30 households, which varied depending on the geographical characteristics of the area (Fig. 2).

To reduce cholera transmission within the case clusters, the appropriate water and hygiene interventions were conducted depending on the transmission context (e.g., case households, public places in the community) [24].

The following response activities were carried out at case residences, close neighboring residences (≤50 m from a case household), and high-risk peripheral residences (> 50 m from a case household) within case clusters:

- Household drinking water was systematically chlorinated everyday over a 14-day period using either water purification tablets (sodium dichloroisocyanurate, 7 mg) or 1% chlorine solution. To treat water of low turbidity (< 5 NTU), 2 ml of 1% chlorine stock solution was added to 10 l of water (final concentration 2 mg/L). To treat turbid water (> 5 NTU), a jar test was used to determine the treatment necessary [19, 20].
- Each household was provided with a household hygiene kit, containing soap, a 20-l water storage container and ready-to-use chlorine for disinfection of drinking water containers [22, 23]. Water storage containers were distributed together with instructions to safely store household drinking water [25].
- To enhance health awareness and encourage safe practices, hygiene and health messages were delivered to the household members [19, 21].
- For cholera case residences only, surfaces likely to be contaminated with vomit or diarrhea from a cholera patient were disinfected with a 0.2% chlorine solution [21] within less than 72 h after patient registration at the CTC.

The activities carried out at in public places in the community within case clusters, over the course of 14 days, are described below. Additional details concerning the water and hygiene interventions in each health zone are displayed in Table 1.

- Water bladders (10-m^3^) were installed in public places. The water bladders are self-supporting closed flexible tanks composed of a coated polyester mesh, which are used to store drinking water for distribution (Labaronne Citaf, Pont-Eveque, France; https://www.labaronne-citaf.com/products/self-supporting-closed-flexible-tank/). Water bladders were installed in neighborhoods of high population density without a source of safe drinking water nearby, neighborhoods that were not covered by the public water network and crowded public areas [19, 26]. Two water bladders were installed in Limeté near the CTC, where they also served to provide safe drinking water to the local population, and one water bladder was installed in Kingabwa. The water bladders were refilled every 48 to 72 h by national water company tankers (Regideso). The residual chlorine levels were verified prior to distribution.
- Fixed water chlorination points (bucket chlorination) were installed in public places in the health zones most affected early during the outbreak: Binza Météo and Kintambo.
- Handwashing points were installed in public places in Binza Météo and Kintambo.
- Hygiene education messages were disseminated to the community to promote health-seeking behaviors and protection mechanisms via health promotion campaigns in public places (i.e., markets, schools, transport stations, water points). Messages were communicated via radio, TV, posters and town criers, and topics included the modes of transmission, water treatment, and the importance of reporting cases of severe diarrhea [20].
- Public health rules were enforced by the local authorities - swimming in waterbodies (e.g., lakes, rivers, streams) was banned during the 14-day period [27].

**Table 1:** Response intervention details per health zone

Health zone	Details of water and hygiene interventions in the community			
	*Intervention duration*	*Number of water bladders*	*Number of fixed water chlorination points*	*Number of handwashing points*
Binza Météo	60 days	0	15	4
Limeté	30 days	2	0	0
Kintambo	30 days	0	2	3
Kingabwa	30 days	1	0	0
Bumbu	30 days	0	0	0

Field response teams consisted of a supervisor, two hygiene promotion educators (a crier and a door-to-door educator), four team members who performed water chlorination (two for fixed sites and two for door-to-door household visits), two team members who performed home disinfection and two attendants at handwashing points. Each team covered at least two 30-household grid units. The number of personnel involved per intervention type in each health zone is detailed in Table 2.

**Table 2:** Number of personnel involved per intervention type for each health zone

Health zone	Number of personnel per intervention type and role				
	*Hygiene promotion*	*Water chlorination*	*Home disinfection*	*Supervision*	*Total Personnel*
Binza Météo	135	60	42	20	257
Limeté	8	10	10	4	32
Kintambo	40	8	10	5	63
Kingabwa	18	17	10	5	50
Bumbu	40	40	30	8	118
Total	241	135	102	42	520

### Additional response activities conducted in case clusters

In parallel with water and hygiene activities, active case search was carried out in the community, prioritizing the immediate entourage of probable and confirmed cases identified or treated at the CTC [20]. Chemoprophylaxis of all immediate contacts of cholera cases was also conducted during household visits to provide short-term protection against infection [28, 29]. Adults received a single dose of doxycycline (300 mg), pregnant women received a single dose of ciprofloxacin (1 g) and children received a single dose of ciprofloxacin (20–30 mg/kg) [19].

### Ethics

Ethics approval was not required for this study because cholera surveillance and response are covered by national public health laws as an integral part of the public health mandate of the DRC Ministry of Health.

## Results

### Spatiotemporal assessment of the cholera outbreak in Kinshasa during the 2017–2018 period

**Fig. 3:**
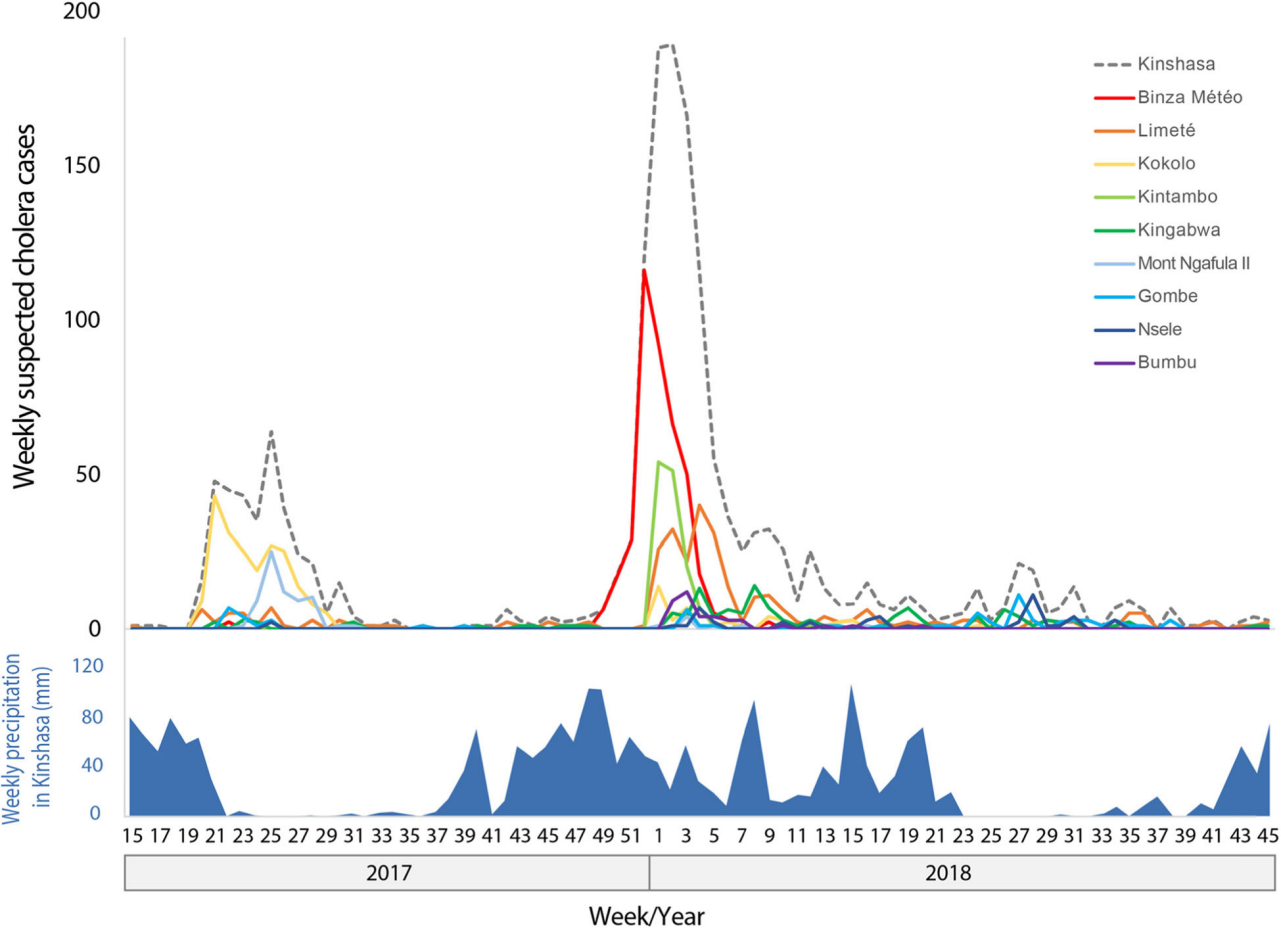
Epidemic curves of the cholera outbreak in Kinshasa and corresponding weekly precipitation levels. The epidemic curve and weekly precipitation levels cover week 15 of 2017 to week 45 of 2018. The top panel displays weekly cholera case numbers in the entire city (dashed line) as well as heavily-affected health zones, which are color-coded and ordered based on cumulative number of cholera cases during the 2017–2018 period (up to week 45, 2018) as displayed in Additional file 1. The bottom panel displays the corresponding estimated weekly precipitation levels in Kinshasa (mm)

In the context of the largest cholera epidemic in the DRC since 1994, a total of 1712 suspected cholera cases, including 53 deaths (CFR 3.1%), were reported in Kinshasa from week 1 of 2017 to week 45 of 2018. *Vibrio cholerae* O1 Inaba was identified as the causative bacterium. During this period, the first outbreak in Kinshasa occurred from mid-May to late-August 2017 and remained primarily confined to a military camp in Kokolo (Fig. 3), a closed environment of high population density (220 cases, CFR 6.4%).

The second and main outbreak began on November 25, 2017 [30]. This outbreak spread quickly throughout Kinshasa, with cases first reported in densely inhabited Camp Luka in Binza Météo Health zone (red line in the epidemic curve), followed by Limeté during the last week of 2017 (orange line), and Kintambo during early-January 2018 (light green line) (Fig. 3).

The outbreak in Kinshasa peaked during the first 2 weeks of January 2018, when 188 and 189 suspected cases were reported per week, respectively. By the first week of January, the cholera outbreak had already spread throughout Kinshasa, affecting the health zones of Binza Météo, Kintambo, Bandalungua, Mont Ngafula II, Kokolo and Limeté. Cases were also reported in Bumbu during the second week of 2018 (purple line in Fig. 3). Nine other health zones in the city reported cholera cases by mid-January, and an additional 11 health zones reported cholera cases by late-January.

**Fig. 4:**
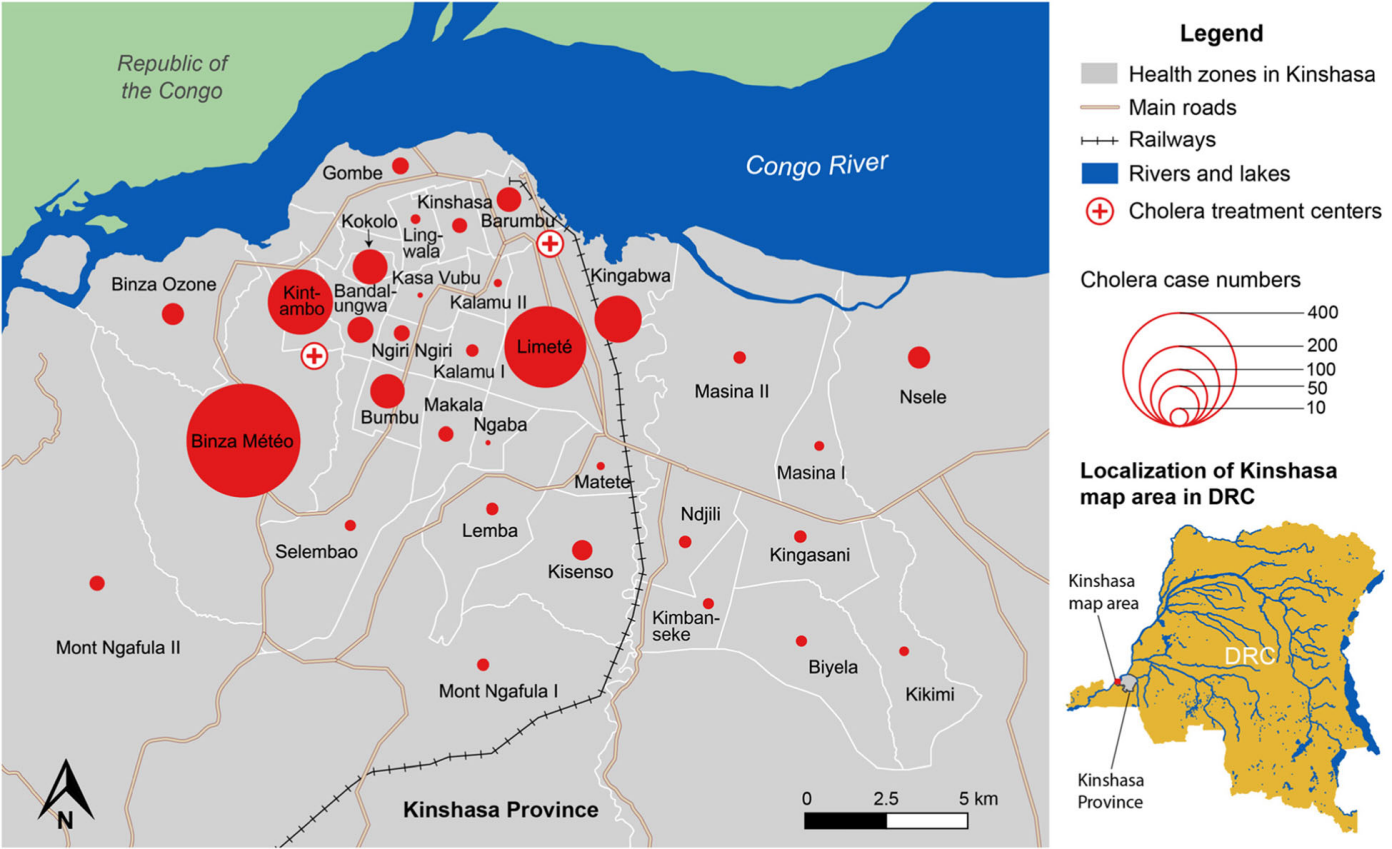
Spatial localization of all cholera cases per health zone in Kinshasa from November 2017 to March 2018. The red circles represent the number of cumulative cholera case numbers (suspected and confirmed) in each health zone during the five-month period. The only areas not represented on the map are the large health zones located in the east of Kinshasa Province, Maluku II and Maluku I, which reported seven and 21 cases, respectively, during the five-month period. Health zones, main roads, railroads and waterbodies in Kinshasa are indicated on the map. The locations of the CTCs in Binza Météo (Camp Luka) and Limeté (Pakadjuma) are also indicated. Neighboring Republic of the Congo is shown in green. Localization of Kinshasa Province (gray) and the Kinshasa map area (red square) are specified on the map of the DRC in the lower right corner

During the main outbreak period from November 2017 to March 2018 (week 45, 2017 - week 13, 2018), a total of 1097 cholera cases, including 11 cholera-related deaths, were reported in Kinshasa. The majority of cases were concentrated in health zones in northwest Kinshasa, less than 10 km from the Congo River. During this period, Binza Météo Health zone reported 37% of all cases (405 cases), followed by Limeté (19%; 208 cases), Kintambo (12%; 134 cases), Kingabwa (6%; 69 cases), Kokolo (3%; 38 cases) and Bumbu (3%; 37 cases). Together, these six health zones reported 81% of all cholera cases in Kinshasa during the five-month period (Fig. 4).

### Reduction in case numbers following implementation of the targeted response strategy

**Fig. 5:**
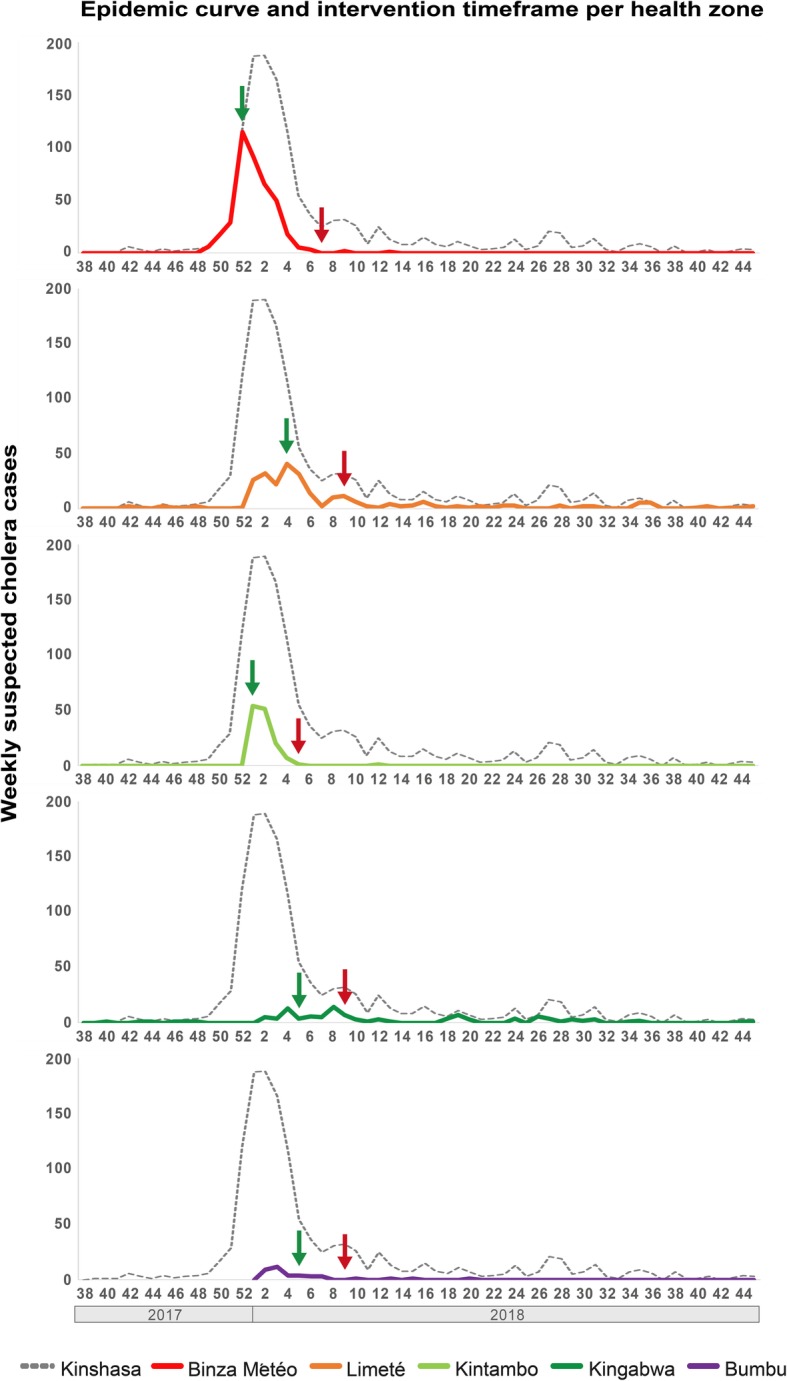
Cholera epidemic curve per targeted health zone and response activity timeframe. Weekly cholera case numbers are shown on the y-axis and epidemic weeks/years are indicated on the x-axis. The start and end points of the response activities in each health zone are shown with green and red arrows, respectively

The response strategy was first implemented in Camp Luka (Binza Météo) during the last week of 2017, when 116 weekly cases were reported in the health zone. Binza Météo was the most affected area and the starting point of the outbreak. In Binza Météo, the weekly number of cases quickly dropped following response implementation, with less than five cases reported per week by early February. As the outbreak spread, the cluster grid response strategy was then implemented in Kintambo during the first week of January, followed by Limeté in late-January and both Kingabwa and Bumbu in early-February. In Kintambo and Limeté, implementation of the response strategy also led to a rapid decrease in cholera cases; both outbreaks were controlled by mid- to late-February (Fig. 5).

We assessed the reduction in weekly cholera case numbers following implementation of the response strategy in the health zones experiencing the largest outbreaks: Binza Météo, Kintambo and Limeté. Two weeks after implementation of the response strategy, the weekly cholera case numbers in the three health zones decreased by 43, 63 and 65%, respectively, compared to starting point weekly case numbers. Four weeks after strategy implementation, the weekly case numbers decreased by 85, 98 and 75%, respectively. Eight weeks after strategy implementation, the weekly case numbers decreased by 100, 100 and 98%, respectively. Considering all cholera cases reported in Kinshasa, the weekly case numbers dropped by 71% 4 weeks after the outbreak peak and by 83% 8 weeks after the peak (Table 3). Kingabwa and Bumbu were not included in the health zone-specific analysis, as weekly cholera case numbers remained below 14 and four, respectively.

**Table 3:** Reduction in cholera case numbers following implementation of the response

	**Number of cases at response starting point**	**Reduction in number of cholera cases (%) compared to the implementation week**			
Health zone	Number of weekly cases during week of response implementation	**After one week**	**After two weeks**	**After four weeks**	**After eight weeks**
Binza Météo	116	20.7%	43.1%	84.5%	100%
Kintambo	54	5.6%	63%	98.1%	100%
Limeté	40	22.5%	65%	75%	97.5%
	Number weekly cases once the response was implemented in two health zones				
Kinshasa Province Total	188	−0.5%	11.7%	70.7%	83%

Overall, the cluster grid response was implemented in five health zones that accounted for 78% of all cases reported in Kinshasa between November 2017 and March 2018. Response activities were initiated in the targeted health zones between 1 to 4 weeks after the first local cases were reported, and the outbreaks were controlled between 3 to 7 weeks after the water supply and hygiene activities were initiated.

## Discussion

In November 2017, a cholera outbreak was reported in Kinshasa, DRC. The outbreak quickly spread throughout the city, affecting 31 of 35 health zones by early-February. The response strategy was implemented during the peak period to rapidly contain the outbreak by targeting five heavily-affected health zones. This strategy targeted case clusters with interventions focused on emergency water supply, household water treatment and safe storage, home disinfection and hygiene promotion. Interventions targeting case households and nearby neighbors were organized using a cluster grid approach. Following implementation of the response strategy, the outbreak in Kinshasa was quickly brought under control. In the three health zones reporting the most cases − Binza Météo, Kintambo and Limeté − the weekly cholera case numbers dropped by an average of 57% by 2 weeks post-response implementation and 86% by 4 weeks post-response implementation. The total weekly case numbers throughout Kinshasa Province dropped by 71% 4 weeks after the outbreak peak and by 83% 8 weeks after the peak.

Previous studies have shown that the risk of cholera infection is significantly higher for household contacts of cholera patients [31], especially during the week after the cholera case seeks treatment [32, 33]. As a result, interventions targeting case households using a variety of response measures have been applied during cholera outbreaks, although little evidence has been published concerning the effectiveness, efficiency or optimal implementation strategy.

Furthermore, cholera risk in urban settings has been shown to increase among nearby neighbors of cholera cases during the initial 5 days following disease onset [34]. The relative risk of infection during the first 3 days has been shown to be 36 times greater within a 50-m radius of a confirmed case, six times greater within a 51- to 100-m radius, and five times greater within a 101- to 150-m radius [34, 35]. A recent micro-simulation model has highlighted the potential impact of case-area targeted interventions in response to cholera outbreaks [36]. The study also found that early intervention was important to rapidly interrupt disease transmission [36]. Response strategies targeting case households and nearby neighbors are especially critical in urban contexts due to the limited availability of resources for non-targeted approaches and explosive potential of outbreaks in overcrowded areas. To ensure a rapid response, early case detection, case confirmation and pre-positioning of response supplies (WASH and case management) are fundamental.

In addition to describing the package of water supply and hygiene interventions carried out during the outbreak response, we also describe the antibiotic prophylaxis of all immediate contacts of cholera cases. Antibiotic prophylaxis was included among emergency control interventions to reduce short-term risk of infection. However, considering the potential risk of bacterial drug resistance, this measure is not recommended by the WHO [28], and we therefore do not aim to promote nor analyze this intervention in our study.

Some study limitations should be noted. First, as we assessed the overall pattern of the outbreak, before and after implementation of the targeted strategy, we cannot establish the effect of the individual interventions in this report. Confounding effects of individual interventions are also difficult to demonstrate; for example, increasing the quantity and quality of available water may also have an effect on improved household hygiene [37]. Second, this study lacks a non-intervention control group because the response strategy was carried out in all health zones experiencing over 3% of cholera cases from November 2017 to March 2018 (with the exception of the closed environment Kokolo Military Camp), and non-intervention would pose major ethical concerns. Third, as this is the first implementation of the cluster grid strategy, further studies are needed to fully ascertain the potential of this approach and refine strategy design. Additional studies should include household surveys to assess WASH indicators and outcome indicators. A cross-sectional survey should be included to assess the improvements made following the strategy. Systematic assessment of water samples should be included to determine changes in water quality.

Overall, our results demonstrate that cholera case numbers rapidly decreased throughout Kinshasa following implementation of the targeted response strategy. Our findings provide valuable lessons from the field for actors and international donors involved in cholera control. To eventually eliminate cholera, it is important to establish long-term solutions to ensure a safe and sustainable drinking water supply and improved sanitation for the population [38–40]. However, until potable water and proper sanitation can be ensured in a sustainable manner in at-risk areas, this response strategy using the cluster grid approach may be adapted to quickly stop cholera transmission in other urban settings.

## Conclusions

During the 2017–2018 period, Kinshasa experienced a cholera outbreak that showed potential to quickly spread throughout the city. The cluster grid response strategy was developed and implemented to rapidly contain the outbreak. The response strategy targeted case clusters in five heavily-affected health zones, in which activities at case residences, nearby neighbors and public places in the community focused on emergency water supply, household water treatment and safe storage, home disinfection and hygiene promotion. Following implementation of the response, the outbreak in Kinshasa was quickly brought under control. In health zones experiencing the largest outbreaks - Binza Météo, Kintambo and Limeté - the weekly cholera case numbers decreased on average by 57% 2 weeks post-response implementation and by 86% 4 weeks post-response implementation. A similar approach may be adapted to quickly stop cholera transmission in other urban settings.

## Supplementary information

**Additional file 1..** Cholera case numbers and case fatality rates in highly affected health zones of Kinshasa.

## Abbreviations

CFR: Case fatality rate
CHIRPS: Climate Hazards Group InfraRed Precipitation with Station
CTC: Cholera treatment center
DRC: Democratic Republic of the Congo
WASH: Water, sanitation and hygiene
WHO: World Health Organization
