# Supplementary material for: Description of the targeted water supply and hygiene response strategy implemented during the cholera outbreak of 2017–2018 in Kinshasa, DRC
Source: BMC Infect Dis. 2020 Mar 18;20:226. doi: 10.1186/s12879-020-4916-0 (PMC7079479; doi:10.1186/s12879-020-4916-0)
Supplement: Supplementary file 1 — Additional file 1.. Cholera case numbers and case fatality rates in highly affected health zones of Kinshasa. [file 12879_2020_4916_MOESM1_ESM.docx]

# Additional files

**Additional file 1. Cholera case numbers and case fatality rates in highly affected health zones of Kinshasa.**

| **Health zone** | **Total cases** | **Proportion of cases (%)** | **Total deaths** | **CFR (%)** |
| --- | --- | --- | --- | --- |
| Binza Météo | 407 | 23.8 | 8 | 2.0 |
| Limeté | 309 | 18.0 | 7 | 2.3 |
| Kokolo | 251 | 14.7 | 14 | 5.6 |
| Kintambo | 143 | 8.4 | 2 | 1.4 |
| Kingabwa | 124 | 7.2 | 1 | 0.8 |
| Mont Ngafula II | 76 | 4.4 | 2 | 2.6 |
| Gombe | 69 | 4.0 | 2 | 2.9 |
| Nsele | 47 | 2.7 | 5 | 10.6 |
| Bumbu | 41 | 2.4 | 1 | 2.4 |
| Maluku I | 38 | 2.2 | 2 | 5.3 |
| Barumbu | 23 | 1.3 | 0 | 0 |
| Bandalungwa | 22 | 1.3 | 0 | 0 |
| Kisenso | 18 | 1.1 | 2 | 11.1 |
| Binza Ozone | 17 | 1.0 | 0 | 0 |
| Mont Ngafula I | 13 | 0.8 | 2 | 15.4 |
| Masina II | 12 | 0.7 | 1 | 8.3 |

*The 16 health zones listed in the table represent 94% of all suspected cases reported in Kinshasa between week 1 of 2017 to week 45 of 2018. The remaining affected health zones reported less than 10 cases each.*
